# New insights into the influence of temperature on axonal transport and function in myelinated regions of Schwann cells

**DOI:** 10.1016/j.isci.2025.113467

**Published:** 2025-08-28

**Authors:** Chih-Wei Zeng, Yasuhiro Kamei

**Affiliations:** 1 Department of Neuroscience, University of Texas Southwestern Medical Center, Dallas, TX 75390, USA; 2 Department of Basic Biology, Graduate University for Advanced Studies (SOKENDAI), Okazaki Aichi 444-8585, Japan; 3 Optics and Imaging Facility, Trans-Scale Biology Center, National Institute for Basic Biology, Okazaki, Aichi 444-8585, Japan

**Keywords:** Neuroscience, Techniques in neuroscience, Cell biology

## Abstract

Temperature within the range of 15°C–37°C plays a pivotal role in modulating cellular processes and is essential for understanding the complex mechanisms underlying axonal transport and function in myelinated regions of Schwann cells (SCs). This review presents a comprehensive overview of the current knowledge on the impact of temperature on various aspects of axonal function, including saltatory conduction, ion channel activity, molecular motor dynamics, and Schwann cell function. We also delve into the potential implications of these findings in the context of neurological disorders and their treatment. The temperature-dependent nature of saltatory conduction and action potential propagation in myelinated axons is of particular interest, as it directly affects the efficiency of nerve signal transmission. Additionally, the activity of ion channels in the nodes of Ranvier is subject to modulation by temperature, further emphasizing the importance of understanding temperature’s influence on neuronal function. This review concludes with a discussion of various unresolved questions in the field, and ideas are suggested for future research. Studying the precise molecular mechanisms underlying the temperature-dependent regulation of ion channels, molecular motors, and cytoskeletal components may lead to the development of novel strategies for the diagnosis and treatment of neurological disorders, which are commonly observed in demyelinating diseases and hereditary neuropathies. A deeper understanding of the role of temperature in neuronal function has the potential to significantly advance our knowledge of thermoregulation and neurologic function, ultimately leading to breakthroughs in the diagnosis and treatment of various neurological disorders.

## Graphical abstract

**Figure:**
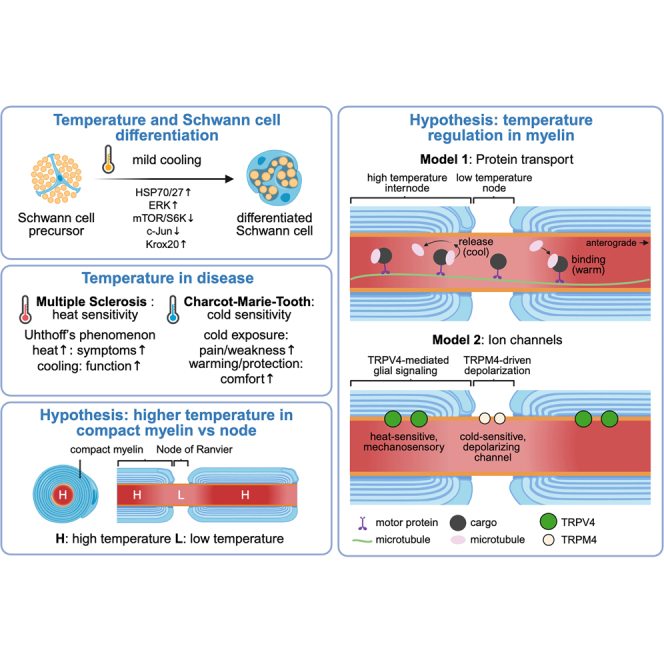

## Introduction

The nervous system, a highly complex and dynamic network, is essential for facilitating communication and orchestrating a myriad of functions and responses within an organism. Myelinated axons play a crucial role in ensuring rapid and efficient transmission of electrical signals, which is necessary for the proper functioning of the nervous system. These axons are formed by specialized glial cells, specifically oligodendrocytes and Schwann cells (SCs), that generate myelin sheaths to envelop axons in the central and peripheral nervous systems (CNS and PNS, respectively).^1^^,^^2^ While the significance of myelinated axons in neural function is well established, the precise influence of temperature on axonal transport and function in these cells remains a subject that requires further investigation.

Recent studies have shed light on the temperature-dependent nature of saltatory conduction and the propagation of action potentials in myelinated axons.^3^^,^^4^ These findings highlight the importance of temperature in modulating the activity of ionic channels in the nodes of Ranvier (NoR), which directly impacts the efficiency of nerve signal transmission.^5^^,^^6^ Furthermore, temperature has been identified as a key factor in the dynamics of molecular motors involved in axonal transport, as both cytoplasmic dynein and kinesin exhibit temperature-dependent characteristics.^7^^,^^8^ Temperature has also been found to influence a wide range of biophysical processes, including biomolecule diffusion, enzymatic activity, and heat shock-induced gene expression.^9^^,^^10^^,^^11^^,^^12^ This evidence suggests that temperature may play a crucial role in determining the stability and function of the intricate network of proteins, linkers, molecular motors, and cytoskeletal components within neurons. However, until recently, the absence of suitable tools for measuring temperature at the micrometer scale *in vivo* has limited our understanding of these processes. The development of genetically encoded ratiometric fluorescent temperature indicators (gTEMP; or new version, B-gTEMP) has enabled researchers to measure temperature at the micrometer scale *in vivo*, opening new avenues for investigating the role of temperature in neural function.^13^

Understanding how temperature influences neuronal function is critical for unraveling the complexities of nervous system physiology and pathology. This review provides a comprehensive examination of the temperature-dependent regulation of axonal transport and function in myelinated regions of SCs. Specifically, we explore how temperature affects saltatory conduction, axonal transport, and the stability of protein networks within neurons. We discuss potential mechanisms by which temperature modulates key cellular components, including molecular motors, ion channels, and other elements that contribute to the functionality of the nervous system. By synthesizing current research, we aim to highlight the intricate interplay between temperature and neuronal activity in these vital cellular structures. Additionally, we identify critical gaps in knowledge and emphasize the need for further investigation into this underexplored area. A deeper understanding of temperature-mediated regulation in myelinated axons could provide valuable insights into neurological disorders associated with aberrant myelination or impaired axonal transport. To advance this field, we advocate for interdisciplinary collaboration, integrating expertise from neuroscience, biophysics, biochemistry, and computational modeling. By fostering such collaborative efforts, we hope to facilitate the development of novel diagnostic and therapeutic strategies for neurological conditions linked to temperature-sensitive disruptions in axonal function.

## Temperature and neuronal development

Recent studies have also highlighted the potential impact of temperature on the development and plasticity of the synapse, which is the site of communication between neurons. Temperature-sensitive regulation of the molecular mechanisms involved in synaptic plasticity, such as long-term potentiation and long-term depression, has been reported, indicating that the optimal temperature range is essential for the efficient functioning of synapses.^14^^,^^15^ Moreover, temperature fluctuations during development have been shown to have significant consequences for the formation of neuronal circuits and the establishment of functional connectivity in the developing nervous system.^16^ The exact mechanisms by which temperature influences these processes remain unclear, highlighting the need for further research in this area. Additionally, the impact of temperature on neuronal metabolism, including glycolysis, oxidative phosphorylation, and ATP production, has been studied. Studies have suggested that changes in temperature can influence the metabolic processes within neurons, ultimately affecting neuronal function and survival.^17^^,^^18^ Temperature-dependent regulation of metabolic processes is of particular interest in the context of neurological disorders, such as Alzheimer’s disease and Parkinson’s disease, where disruptions in neuronal metabolism are commonly observed.^19^^,^^20^ Understanding the precise molecular mechanisms underlying the temperature-dependent regulation of neuronal metabolism could offer novel therapeutic targets for these diseases.

In conclusion, temperature has a significant impact on a wide range of biophysical processes within neurons, including saltatory conduction, ion channel activity, molecular motor dynamics, myelination, synaptic plasticity, neuronal development, and metabolism. The intricate relationship between temperature and neuronal activity is complex, and further research is needed to unravel the precise molecular mechanisms underlying this phenomenon. Addressing the remaining challenges in this field necessitates interdisciplinary collaboration among researchers from diverse fields, which could ultimately lead to significant breakthroughs in our understanding of thermoregulation and neurologic function.

## Temperature-dependent regulation of saltatory conduction and ionic channels

The influence of temperature on saltatory conduction and action potential propagation in myelinated axons is crucial for the proper functioning of the nervous system.^3^^,^^4^ The activity of ion channels within the NoR is particularly sensitive to temperature changes, which can directly impact the efficiency of nerve signal transmission.^5^^,^^6^ One example of temperature-mediated modulation of ion channels is the activity of TRPM4 channels, which are known to be activated by cooling and preferentially accumulate in the NoR rather than myelinated regions,^6^ suggesting a spatially distinct regulatory mechanism tied to local temperature gradients. Several studies have demonstrated that TRPM4 channel activity is strongly temperature-dependent in the warm physiological range (∼15°C–37°C).^21^^,^^22^ Compared to many other ion channels with modest Q_10_ values of ∼2–4, TRPM4 exhibits unusually steep thermal sensitivity, with reported Q_10_ values around 8–10.^23^ Warming not only increases current amplitude but also shifts the voltage dependence of activation (V_1_/_2_) to more negative values, approximately −25 mV per 10°C, making the channel more likely to open under physiological conditions.^23^^,^^24^ This thermal shift in gating occurs without significant changes in Ca^2+^ affinity. At higher temperatures, TRPM4 also inactivates more rapidly at negative voltages, contributing to enhanced outward rectification (Table 1). Structural analysis has further identified Glu396 as a key intracellular site responsible for this temperature sensing; mutation of this residue abolishes the heat-induced enhancement of TRPM4 activity.^21^

**Table 1:** Temperature dependence of TRPM4 channel activity: Key studies

Experimental system	Temperature range	Key findings	Reference
HEK293 cells expressing TRPM4 (patch-clamp)	15°C–35°C (warm range)	- Q_10_ ≈ 8.5 between 15°C and 25°C (at +25 mV) - heat shifts voltage activation ∼−25 mV per 10°C - Ca^2+^ sensitivity remains stable with temp	Huffer et al., 2024^22^
tsA201/HEK cells expressing human TRPM4 (whole-cell patch)	∼22°C (room temp) vs. 37°C (physiological)	- large increase in current amplitude at 37°C - faster inactivation of inward currents - E396A mutation abolishes heat responsiveness	Hu et al., 2024^21^
Isolated arterial smooth muscle (myography; TRPM4 in native cells)	∼25°C–40°C (perfusion warming)	- Q_10_ ≈ 11–20 for TRPM4-mediated tone - half-maximal activation near 33°C - TRPM4 contributes to thermal vasoconstriction	Phan et al., 2025^25^

In addition to TRPM4, temperature-dependent gating is a common feature among many ion channels, including voltage-gated sodium (Nav) and potassium (Kv) channels.^26^ These channels play a critical role in the generation and propagation of action potentials. Temperature changes can modulate the kinetics of Nav channel activation and inactivation, impacting both the amplitude and duration of action potentials.^27^ Similarly, temperature fluctuations can influence the activity of Kv channels, altering the repolarization phase of the action potential and subsequently affecting the efficiency of nerve signal transmission.^28^ Moreover, temperature can affect the function of other ion channels, such as hyperpolarization-activated cyclic nucleotide-gated (HCN) channels, which contribute to the regulation of neuronal excitability.^29^ Temperature changes can modulate HCN channel activity by altering the voltage-dependence of activation, directly affecting the resting membrane potential and firing properties of neurons.^30^

Despite these findings, the precise molecular mechanisms through which temperature affects ion channel behavior, especially within the confined architecture of NoRs, remain incompletely understood. Future studies are needed to dissect how local temperature gradients influence ion channel function and, by extension, saltatory conduction. These findings could yield novel perspectives on the pathophysiology of temperature-sensitive neurological disorders, such as multiple sclerosis and certain peripheral neuropathies,^31^^,^^32^ and may support the development of targeted therapies aimed at modulating thermosensitive ion channels.

## Temperature influence on molecular motors and axonal transport dynamics

Cytoplasmic dynein and kinesin, two microtubule-based molecular motors with distinct structural and evolutionary backgrounds, display temperature-dependent properties that significantly impact axonal transport processes.^7^^,^^8^ Both dynein, which moves toward the minus end of microtubules, and kinesin, which primarily moves toward the plus end, exhibit comparable unloaded velocities at temperatures above the restrictive threshold for fast axonal transport (below 15°C).^8^^,^^33^ Gaining a deeper understanding of the precise mechanisms underlying the temperature-mediated modulation of these molecular motors’ dynamics is crucial for elucidating the role of temperature in axonal transport and overall neuronal function.

Temperature affects the biophysical properties of molecular motors, altering their motor domain conformation, as well as ATP hydrolysis and release rates.^34^^,^^35^ These changes can lead to variations in the force generation, processivity, and velocity of dynein and kinesin, ultimately influencing the efficiency and reliability of axonal transport.^36^^,^^37^ Furthermore, temperature may affect the interactions between molecular motors and their cargo, with potential consequences for cargo binding, transport, and release.^38^ For example, the binding affinity of kinesin to its cargo can be influenced by temperature, which could lead to changes in cargo selectivity and transport efficiency.^39^ Temperature may also modulate the coordination between multiple molecular motors, which often work together to transport cargos along microtubules.^40^^,^^41^ As different motor types can exhibit distinct temperature sensitivities, temperature fluctuations may alter the balance of forces exerted by opposing motor teams, potentially affecting the directionality and efficiency of cargo transport.^42^ Supporting this, recent comparative studies have quantified how axonal transport velocities vary across neuronal systems under different temperature conditions (Table 2). These data reveal consistent Q_10_ values for transport rates across species, with clear evidence that dynein becomes a limiting factor for transport below ∼15°C, contributing to transport failure in cold environments. Interestingly, the data also highlight species-specific thresholds for cold block and differential transport velocities between adrenergic and dopaminergic axons, emphasizing that both motor identity and neuronal subtype influence thermal sensitivity. *In vitro* single-molecule assays further confirm that kinesin exhibits a relatively smooth increase in motility with temperature, while dynein shows a sharper drop in function below 15°C, consistent with its role in cold-induced transport arrest observed *in vivo*. These observations underscore the importance of both molecular and systemic context in determining temperature sensitivity of axonal transport. Lastly, it is important to consider the potential influence of temperature on the stability and organization of the microtubule tracks themselves, as temperature-induced changes in microtubule dynamics and post-translational modifications could further impact motor function and axonal transport.^46^^,^^47^

**Table 2:** Temperature-dependent motor protein velocities in neuronal systems

System type	Temperature range	Motor/cargo	Velocity data	Physiological relevance	Reference
*Ex vivo* garfish olfactory nerve (axons)	10°C–28°C	fast transport (anterograde)	- 53 mm/day at 10°C - 249 mm/day at 25°C - ∼410 mm/day at 37°C	- linear increase with temperature - high velocity (∼4.7 μm/s) at 37°C	Yadav and Kunwar, 2021^35^
*Ex vivo* frog sciatic nerve (radiolabeled proteins)	5.5°C–28°C	fast transport (anterograde)	- 32–290 mm/day over range - extrapolated ∼400 mm/day at 37°C - Q_10_ ∼3.4–2.3	- transport persists at low temp - matches warm-blooded velocity at 37°C	Edstro and Hanson, 1973^43^
*Ex vivo* rabbit sciatic nerve (stop-flow assay)	13°C–42°C	dopamine-β-hydroxylase	- velocity = 0.546·(1.09)ˆT - ∼0.4 mm/h at 10°C - ∼12.8 mm/h at 37°C - Q_10_ ≈ 2.3	- cold block below ∼13°C - transport reaches ∼3.6 μm/s at 37°C	Cosens et al., 1976^44^
*Ex vivo* rabbit and frog sciatic nerves (adrenergic axons)	10°C–38°C	fast transport (DBH)	- exponential increase - transport ceases below ∼13°C (rabbit), ∼10°C (frog)	- species-specific cold block - linked failure to dynein sensitivity at low T	Brimijoin et al., 1979^45^
*In vitro* single-molecule motility (purified mammalian motors on MTs)	5°C–37°C	kinesin vs. dynein	- kinesin: smooth increase - dynein: sharp drop below ∼15°C - Q_10_ breaks at 15°C	- dynein likely cause of cold-block - explains transport failure <15°C *in vivo*	Hong et al., 2016^8^

In conclusion, Table 2 provides an integrated overview of temperature-dependent motor protein velocities across experimental systems, supporting the notion that motor activity is strongly influenced by thermal fluctuations. Further research is needed to elucidate the complex interplay between temperature and molecular motor dynamics in the context of axonal transport. This knowledge could not only improve our understanding of the fundamental processes underlying neuronal function but also inform the development of therapeutic strategies targeting temperature-sensitive molecular motors in the treatment of neurodegenerative disorders and other temperature-sensitive neurological conditions.

## Temperature influence on myelination and Schwann cell function

SCs are pivotal in the myelination process, which is essential for the optimal functioning of the nervous system. Recent research has indicated that temperature may have a significant impact on the differentiation, proliferation, and survival of SCs.^48^^,^^49^^,^^50^ For instance, it has been demonstrated that decreased temperature can facilitate SC differentiation and myelin formation *in vitro.*^51^ Additionally, temperature variations have been observed to affect the survival of SCs in culture, with moderate hypothermia enhancing cell survival, while hyperthermia leads to cell death.^48^

Investigating the mechanisms responsible for these temperature-dependent effects on SC function is crucial for understanding their implications on myelination and overall neuronal function *in vivo*. Temperature may regulate the expression and activity of various signaling pathways, transcription factors, and other molecules involved in SC differentiation, proliferation, and myelination.^52^^,^^53^ For example, temperature-sensitive signaling pathways, such as those mediated by neurotrophins or extracellular matrix molecules, could be involved in modulating SC responses to temperature changes.^54^^,^^55^ Moreover, temperature can influence the biophysical properties of the lipids and proteins that make up the myelin sheath, potentially affecting its stability, structure, and function.^56^^,^^57^ Changes in temperature may also alter the dynamics of myelin membrane assembly, trafficking, and turnover, which are essential for maintaining myelin integrity and adapting to changes in neuronal activity.^58^^,^^59^ Furthermore, temperature-dependent effects on SC function could have implications for the interplay between SCs and other glial cells, neurons, and immune cells within the nervous system.^60^ For instance, temperature may modulate SC interactions with other cell types, affecting processes such as axon-SC communication, immune cell recruitment, and the activation of repair mechanisms following injury.^61^^,^^62^

In conclusion, uncovering the complex interplay between temperature and SC function is vital for a comprehensive understanding of the role of temperature in myelination and neuronal function. This knowledge could contribute to the development of novel therapeutic strategies targeting temperature-sensitive pathways and processes in SCs, with potential applications in the treatment of demyelinating diseases and other neurological disorders.

## Effects of reduced temperature on Schwann cell differentiation and key pathways

### Lowered temperature enhances Schwann cell differentiation *in vitro*

*In vitro* studies indicate that a mild reduction in culture temperature can promote SC differentiation. For example, culturing precursor cells at 35°C (vs. the standard 37°C) slowed cell proliferation but significantly increased SC differentiation markers. In one study using mesenchymal stem cells induced toward a Schwann-like phenotype, the lower temperature led to higher expression of glial and myelin-related genes (S100, p75NTR, and GFAP) and myelin proteins (P0 and MBP).^63^ This suggests that cooler conditions favor the transition to a mature, myelin-producing SC state, possibly by inducing cell-cycle exit (a prerequisite for myelination) and activating pro-differentiation programs.

### Heat-shock proteins, HSP70 and HSP27, in mild cold stress

Reduced temperature can act as a mild cellular stress, engaging heat shock protein pathways. HSP70 and HSP27, in particular, are molecular chaperones that SCs utilize for protection and differentiation under stress:

6.2.1 HSP70: Elevating HSP70 levels has been shown to promote myelination and prevent SC dedifferentiation. Notably, HSP70 induction can counteract demyelinating signals by targeting c-Jun (a myelination inhibitor) for degradation.^64^ In SC-neuron co-cultures, activation of the heat-shock response (e.g., via HSP90 inhibitors) increased HSP70 and blocked the induction of c-Jun, thereby protecting myelin segments.^65^ C-Jun is a stress-induced transcription factor that serves as a negative regulator of myelination, it rises during injury or stress and drives SCs into a repair (non-myelinating) phenotype.^52^ By enhancing proteasomal clearance of c-Jun, HSP70 effectively lifts this brake on differentiation, allowing myelin genes to be expressed.^64^

6.2.2 HSP27: This small heat shock protein is also linked to SC stress responses and differentiation. HSP27 is strongly induced in SCs after nerve injury, in concert with the JNK/c-Jun pathway.^66^ The retrograde activation of JNK and c-Jun in injured neurons leads to SC expression of HSP27, which helps reprogram SCs into repair-supportive cells.^64^ HSP27 accumulation in distal nerve segments correlates with SCs that support regenerating axons, partly by stabilizing cytoskeletal elements and suppressing apoptosis.^67^ Under non-injury conditions such as mild hypothermia, HSP27 may also be upregulated to some extent as part of a general stress response. Importantly, HSP27 can modulate pro-survival signaling, it activates Akt (PKB), a key kinase upstream of mTOR.^68^ By enhancing Akt activity, HSP27 could help maintain the PI3K/Akt/mTOR pathway even when overall metabolism is slowed, thereby supporting SC viability and growth signals. In summary, mild cold-induced HSPs likely protect SCs and bias them toward differentiation: inducing HSP70/27 improves myelination outcomes.

### MAPK/ERK signaling under reduced temperature

The ERK1/2 MAPK pathway is a central driver of SC differentiation and myelination. During normal development, growth factor signaling through ERK triggers the myelination program in SCs. Genetic studies show that ERK1/2 activation is required for SCs to upregulate myelin genes: sustained ERK/MAPK activity promotes SC differentiation, whereas loss of ERK halts myelination (SCs fail to elevate myelin regulators such as Krox-20).^69^ At reduced temperature, cellular signaling kinetics are generally slower; however, the differentiation observed at 35°C suggests that ERK signaling remains sufficiently active (or perhaps prolonged) to drive the myelination program. It is known that ERK can function somewhat independently of metabolic rate to initiate differentiation. In fact, ERK/MAPK plays a primary role in initiating SC myelination, distinct from mTOR signaling.^48^ In summary, ERK signaling remains the key pro-myelination pathway and is likely still operative or even tuned in timing under mild cold, ensuring that SCs can enter the myelinating program.

### PI3K/Akt/mTOR/S6K pathway and metabolic signaling

The PI3K-Akt-mTORC1 pathway is another major regulator of SC development, mainly controlling growth and myelin production. mTORC1 activity promotes protein synthesis and myelin sheath growth (e.g., increasing myelin thickness), partly through downstream effectors such as S6K (ribosomal S6 kinase). Unlike ERK, mTOR activity is highly sensitive to cellular metabolic state, a drop in temperature generally reduces ATP turnover and can dampen mTOR/S6K signaling.^70^ Experimental data indicate that mTOR is not absolutely required to begin myelination, but it is needed to achieve full myelin growth. Specifically, ERK and mTOR have distinct but cooperative roles: ERK drives the onset of SC differentiation, whereas mTOR mainly amplifies myelin production and sheath thickness.^71^ In support, a study in developing nerves found that excessively high mTORC1 activity can actually impede SC differentiation by suppressing Krox-20 expression.^72^ In normal development, mTORC1 must be downregulated at the proper time to allow myelination to start.^73^ Conversely, when mTORC1 was genetically inhibited in SCs, Krox-20 levels rose and SCs still upregulated myelin genes (though myelin protein output was somewhat reduced).^74^ In other words, hyperactive mTOR can delay the myelination switch, whereas restrained mTOR permits the pro-differentiation transcription factors to elevate.^70^ Overall, a reduced temperature shifts SC signaling toward differentiation over growth: ERK-driven myelin gene induction proceeds, while mTOR-S6K output is scaled back. This balance can actually be beneficial for initiating maturation, as long as basic trophic support (Akt signaling) is maintained. Notably, HSP27’s activation of Akt^75^ under stress may help keep SCs alive and somewhat metabolically competent even with lower mTOR, thereby supporting eventual myelin formation once conditions normalize.

**Figure 1:**
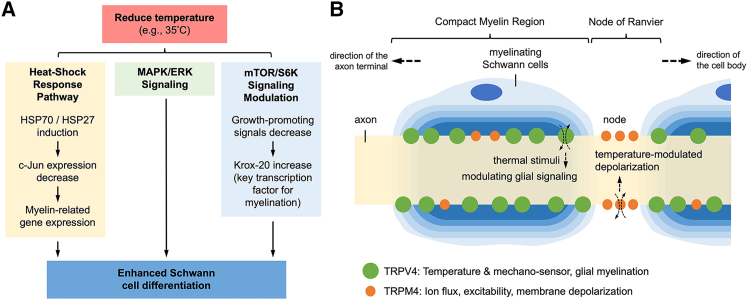
Mechanistic pathways and temperature-sensitive channel distribution involved in Schwann cell differentiation under reduced temperature (A) Schematic representation of signaling pathways activated by mild temperature reduction (e.g., 35°C) that promote Schwann cell (SC) differentiation. Cooler conditions trigger the heat-shock response pathway, inducing HSP70 and HSP27, which suppress c-Jun and enhance myelin-related gene expression. Simultaneously, MAPK/ERK signaling remains active to support pro-myelination transcriptional programs, while mTOR/S6K signaling is downregulated, allowing increased expression of Krox-20, a key transcription factor for SC differentiation. The convergence of these pathways promotes SC maturation. (B) Simplified illustration of a myelinated axon segment showing the spatial distribution of candidate temperature-sensitive ion channels. TRPV4 (green) is enriched in the compact myelin region and associated with temperature/mechanosensation and glial myelination. TRPM4 (orange) is concentrated at the nodes of Ranvier and implicated in ion flux, excitability, and membrane depolarization. These differential localizations suggest complementary roles in regulating SC physiology and axonal conduction under thermal stress.

In summary, mild reductions in temperature (e.g., to ∼35°C) can tilt SCs toward a more differentiated, myelin-ready state *in vitro*. The mechanistic underpinnings involve a coordinated shift in signaling and stress-response pathways: heat-shock proteins (HSP70 and HSP27) are upregulated and help protect the cells and remove inhibitors (such as c-Jun) to myelination; the pro-myelination MAPK/ERK pathway remains active to drive expression of myelin genes; the mTOR/S6K growth pathway is partially subdued, which, somewhat counterintuitively, favors the initiation of differentiation by permitting Krox-20 and myelin proteins to rise (unhindered by overactive growth signals). Key transcription factors respond accordingly: c-Jun levels drop (or its activity is antagonized) as part of the stress response, while Krox-20 and Sox10 are able to accumulate and execute the myelination program (Figure 1A). In parallel, temperature-sensitive ion channels exhibit region-specific distributions along the axon that may further influence Schwann cell physiology and axonal function. TRPV4 is enriched in compact myelin regions and linked to glial mechanosensation and myelination, whereas TRPM4 localizes to NoR, where it modulates membrane excitability and depolarization under thermal stress (Figure 1B).

Future studies may determine whether these channels not only respond to temperature differences but also actively contribute to establishing or maintaining the distinct thermal microenvironments of compact myelin and the NoR. Altogether, the response to reduced temperature recapitulates a pro-differentiation environment, one that slows cell division and activates protective stress pathways, thereby promoting Schwann cell maturation and myelination.

## Temperature and neurological disorders

The impact of temperature on axonal transport and function in myelinated regions of SCs not only affects the understanding of the pathophysiology of various neurological disorders but also has implications for their management and treatment. As mentioned earlier, temperature sensitivity is reported in several demyelinating diseases, such as multiple sclerosis (MS) and certain hereditary neuropathies such as Charcot-Marie-Tooth (CMT) disease. Exploring the role of temperature in these disorders may lead to valuable insights for novel diagnostic and therapeutic strategies. In addition to demyelinating diseases, temperature also plays a role in other neurological conditions. For example, in amyotrophic lateral sclerosis (ALS), a neurodegenerative disease affecting motor neurons, temperature-sensitive misfolding and aggregation of proteins such as superoxide dismutase 1 have been implicated in the disease pathogenesis.^76^^,^^77^ Investigating the effects of temperature on protein aggregation and clearance pathways in neurons could provide a better understanding of the disease mechanisms and potential therapeutic targets for ALS and other proteinopathies.

Temperature sensitivity may also contribute to the pathophysiology of peripheral neuropathies associated with diabetes. Diabetic peripheral neuropathy (DPN) is characterized by the progressive loss of nerve function, often leading to pain, numbness, and weakness in the limbs.^78^ Temperature-dependent changes in ion channel function, axonal transport, and Schwann cell metabolism could exacerbate the detrimental effects of hyperglycemia on nerve function in DPN patients.^79^^,^^80^ Understanding the interplay between temperature and the underlying disease mechanisms in DPN could offer new avenues for the development of targeted therapies. Furthermore, temperature-dependent modulation of neuronal function may be relevant in the context of neurodevelopmental disorders, such as autism spectrum disorder (ASD) and Rett syndrome. Several studies have reported altered temperature sensitivity in individuals with ASD, suggesting that temperature fluctuations might influence the underlying neural mechanisms contributing to the disorder.^81^^,^^82^ Similarly, mutations in the MECP2 gene, which cause Rett syndrome, have been shown to affect temperature sensitivity in animal models, implicating temperature-dependent processes in the pathogenesis of this neurodevelopmental disorder.^83^ Investigating the effects of temperature on neuronal development and function in these disorders may provide insights into their etiology and potential therapeutic interventions.

In conclusion, elucidating the role of temperature in the pathophysiology of various neurological disorders is essential for understanding their underlying disease mechanisms and identifying novel therapeutic targets. Future research should focus on unraveling the complex interplay between temperature and neuronal function in the context of these disorders to advance the development of innovative diagnostic and treatment strategies.

## Thermosensitivity in MS and Charcot-Marie-Tooth disease

Although our primary focus is on the thermosensitivity of peripheral nerves, it is worth noting that nerves do not function in isolation. Temperature shifts can also influence tissues and organs innervated by these nerves, including skeletal muscle, blood vessels, and visceral organs. For example, temperature-dependent changes in vasomotor tone and blood flow can impact peripheral nerve excitability and nutrient delivery.^84^^,^^85^ Similarly, autonomic regulation of gastrointestinal or urogenital function may be impaired in neuropathies with altered thermal sensitivity.^86^ These interactions become especially relevant in neurological disorders characterized by abnormal thermosensation, such as multiple sclerosis (MS) and CMT disease.

### Multiple sclerosis: Heat sensitivity

MS patients commonly experience heat sensitivity: even small increases in core body temperature can temporarily worsen their neurological symptoms, a phenomenon known as Uhthoff’s phenomenon. This has been recognized for over a century and was formally documented in early clinical studies. For example, mild hyperthermia induced in MS patients caused transient exacerbation of neurological deficits.^87^ More recent data confirm the high prevalence of heat sensitivity: a 2022 survey of 757 MS patients reported that 87% experienced heat sensitivity (either heat-only or heat-and-cold), with 58% being heat-only sensitive and 29% sensitive to both heat and cold.^88^ Patients commonly report that hot weather, exercise, or fever trigger fatigue, weakness, and visual disturbances, hallmarks of Uhthoff’s phenomenon.^87^ Conversely, cooling interventions have been shown to alleviate symptoms. A randomized controlled trial demonstrated that wearing a cooling garment for 1 h led to objective improvements in motor function (timed gait) and visual acuity, while one month of daily cooling therapy reduced patient-reported fatigue.^89^ Additionally, a systematic review concluded that pre-cooling or use of cooling garments significantly improves physical performance and functional capacity in heat-sensitive individuals with MS.^90^ These findings provide strong clinical evidence that MS symptoms worsen with heat but can be effectively mitigated by external cooling strategies.

### Charcot-Marie-Tooth: Cold sensitivity and autonomic dysfunction

In contrast to MS, patients with CMT disease frequently exhibit cold sensitivity and related sensory or autonomic disturbances. Many report intolerance to cold temperatures, which may aggravate stiffness, weakness, or discomfort. In a controlled study, a brief cold stress test induced abnormal cardiovascular responses in CMT patients, including significantly higher heart rate increases and oxygen saturation drops, compared to healthy controls.^91^ These responses reflect the patchy demyelination characteristic of CMT and associated autonomic dysregulation, such as abnormal vasomotor and sweating responses.^92^^,^^93^ CMT also often affects small (thinly myelinated) sensory fibers, leading to reduced thermal sensation in the extremities. For instance, Laurà et al. (2014) reported that 53% of CMT1A patients had elevated cold-detection thresholds, consistent with small-fiber dysfunction.^94^

Together, these studies demonstrate that CMT patients are often thermosensitive to cold, exhibiting both subjective intolerance and measurable physiological responses. Autonomic deficits such as impaired thermoregulation likely contribute to these symptoms and warrant consideration in patient care.

## Technological advances for studying temperature effects on neuronal function

Recent technological advancements have significantly improved our ability to investigate how temperature influences axonal transport, myelination, and neuronal function in myelinated regions of SCs. These innovations allow precise measurement, visualization, and functional analysis of temperature-dependent processes at a previously unattainable resolution. A central hypothesis in our research is that temperature differences between compact myelin and the NoRs regulate axonal transport and neuronal function by modulating molecular motor activity and novel temperature-sensitive proteins. Myelinated regions are hypothesized to have higher temperatures, which may facilitate the activation of specific proteins involved in axonal transport, while NoRs, with lower temperatures, may regulate the function of these proteins to influence intracellular cargo transport and saltatory conduction.

### Temperature gradients in myelinated axons

**Figure 2:**
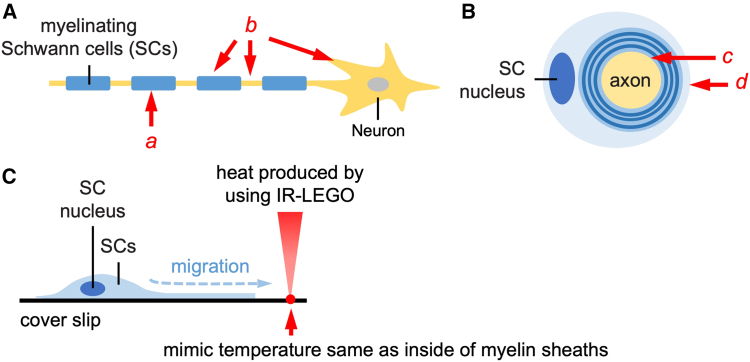
Schematic illustration of myelinating Schwann cells and neuron (A) A simplified diagram showing Schwann cells (SCs) myelinating an axon. The SCs wrap around the axon to form myelin sheaths, which enhance nerve signal conduction. The a (red arrow) indicates the myelin sheath, while b (red arrow) highlights the node of Ranvier, where action potentials are regenerated. (B) Cross-sectional view of a myelinated axon, showing the SC nucleus, myelin sheaths, and axon. The c (red arrow) points to the axon, while d (red arrow) indicates the concentric layers of myelin sheaths wrapped around the axon. (C) Experimental setup illustrating the migration of SCs on a coverslip in response to heat produced using infrared laser light (IR-LEGO). The heating mimics the temperature conditions inside myelin sheaths, promoting SC migration (blue arrow).

Myelinating SCs wrap around axons to form compact myelin, enhancing nerve conduction by allowing action potentials to propagate efficiently through saltatory conduction (Figure 2A). The interaction between SCs and axons contributes to localized temperature differences along the axon, which we hypothesize play a role in regulating axonal function. A cross-sectional view of a myelinated axon reveals that the axon is surrounded by concentric layers of compact myelin, with temperature variations existing between the inner and outer layers (Figure 2B). These temperature gradients may directly impact SC migration and function, as experimentally demonstrated using an infrared laser-evoked gene operator (IR-LEGO) system. This technique enables controlled heat stimulation, mimicking the temperature conditions inside myelin sheaths and promoting SC migration *in vitro* (Figure 2C).

While this model is conceptually supported by functional differences between compact myelin and nodal regions, direct evidence for a micrometer-scale temperature gradient along the axon is currently lacking. No current studies have reported *in vivo* temperature mapping at this resolution, and the physical plausibility of such gradients remains to be rigorously tested. Future work may explore finite-element modeling of axonal^95^ and myelin sheath geometry^96^^,^^97^ using known thermal conductivities of lipids and cytosol to estimate plausible heat retention profiles. In parallel, direct measurements with genetically encoded thermosensors such as gTEMP or B-gTEMP,^13^ targeted to specific axonal compartments, could provide *in situ* validation at subcellular resolution, although technical hurdles remain.

### Experimental approaches to study saltatory conduction

**Figure 3:**
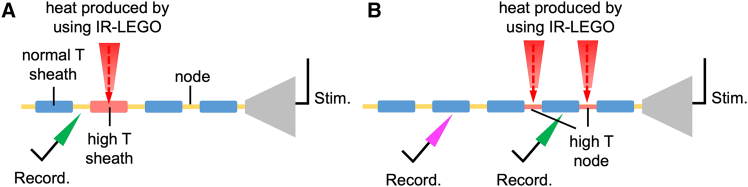
Experimental setup for studying saltatory conduction and sensory behavioral responses (A) Illustration of the experimental design where infrared laser light (IR-LEGO) is used to locally heat a myelin sheath, creating a high-temperature (high T) sheath, while maintaining adjacent normal-temperature (normal T) sheaths. Electrical stimulation (Stim.) is applied at one end, and electrophysiological recordings (Record.) are performed downstream (green arrow) to measure the effects of localized heating on nerve conduction. (B) Experimental setup where heat is applied to both a myelin sheath and a node of Ranvier, generating a high-temperature node. Electrophysiological recordings (Record.) are taken at multiple sites (green and magenta arrows) to assess how heating different regions of the axon affects saltatory conduction.

To explore how temperature influences saltatory conduction, we employ IR-LEGO-mediated heat stimulation at specific regions of myelinated axons. By selectively increasing the temperature of myelin sheaths while maintaining adjacent sheaths at normal temperatures, we can assess the impact of localized heating on nerve conduction using electrophysiological recordings (Figure 3A). Additionally, by applying heat to both a myelin sheath and a NoR, we can analyze how different regions of the axon respond to temperature changes and determine their respective roles in regulating conduction velocity (Figure 3B). This experimental design enables us to investigate whether temperature gradients along myelinated axons enhance or impair action potential propagation.

To ensure physiological relevance, IR-LEGO parameters are typically adjusted to induce local temperature increases of 1°C–3°C,^98^ within the upper range of reported *in vivo* brain and nerve fluctuations under normal and stress conditions.^99^ Laser power settings are carefully calibrated (usually <20 mW) to avoid tissue damage, and brief exposure times (<1 s) help limit unintended heat diffusion.^98^^,^^100^ Previous studies using IR-LEGO in zebrafish and cultured neurons have confirmed the spatial specificity and viability of cells post-stimulation.^101^ However, we acknowledge that exact heat distribution is influenced by tissue composition and optical properties, and future refinement using thermal dyes or computational modeling may help quantify microscale heat diffusion more accurately.

### Proteomic analysis of temperature-dependent axonal transport

**Figure 4:**
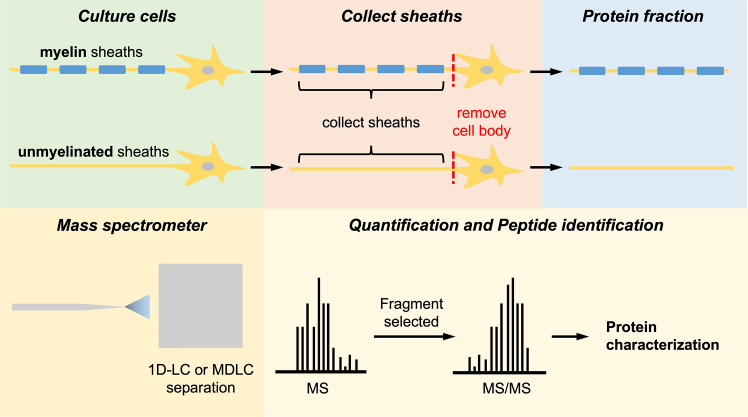
The experimental workflow for proteomic analysis of differentially expressed proteins in myelinated and unmyelinated nerves Neurons are first cultured, with some developing myelin sheaths while others remain unmyelinated. To isolate the sheaths, neuronal cell bodies are removed, leaving behind only the respective sheath fractions for protein extraction. These protein samples are then processed using liquid chromatography (1D-LC or MDLC) before being analyzed by mass spectrometry (MS). Selected peptide fragments undergo tandem mass spectrometry (MS/MS) for further identification and characterization. This approach enables a comparative analysis of the protein composition between myelinated and unmyelinated sheaths, providing insights into the molecular differences underlying myelination.

To identify novel proteins involved in temperature-regulated axonal transport, we use liquid chromatography-mass spectrometry (LC-MS) for proteomic analysis. Neurons are cultured under conditions that allow some axons to develop myelin sheaths while others remain unmyelinated (Figure 4). Using laser microdissection techniques, we selectively isolate myelinated and unmyelinated axon regions, allowing for a direct comparison of their respective proteomes. To minimize contamination from neuronal cell bodies or ganglion-derived proteins, axons are either grown in compartmentalized culture systems that physically separate soma from distal processes^102^ or manually dissected using laser capture microdissection.^103^ After protein extraction, samples are screened for nuclear markers (such as histones or nuclear pore proteins) as negative controls.^104^ These markers are absent or present at negligible levels in purified axonal fractions, supporting the specificity of the isolation protocol. In addition, quality control checks using immunoblotting or LC-MS peptide profiles can further verify that the proteomic data reflect axonal rather than somatic content. This analysis enables the identification of differentially expressed proteins that may contribute to temperature-dependent axonal transport and myelination.

In designing these experiments, careful optimization of culture temperature is critical, as even mild hypothermia or hyperthermia can influence neurite growth, synapse formation, and Schwann cell interactions. Table 3 summarizes recommended temperature ranges for various neural cell culture systems, including primary neurons, Schwann cell-neuron co-cultures, and iPSC-derived neurons, alongside known effects on axonal transport, stress response, and myelination. These guidelines help ensure physiologically relevant conditions are maintained for accurate proteomic comparisons.

**Table 3:** Neural cell culture: Temperature optimization guidelines

Culture type	Optimal temperature	Key observations	Mild hypothermia (∼32°C–34°C)	Mild hyperthermia (∼39°C)	Precautions	Reference
Primary neurons	∼37°C (normothermia)	best survival and neurite growth at 37°C; transport rates drop at 30°C; normal synaptogenesis and proliferation	reduces stress-induced apoptosis; boosts inhibitory synapses; synapses dismantle at ∼18°C–22°C	disrupts presynaptic structure; ≥39°C–41°C triggers HSPs and damage	avoid large shifts; ≥39°C triggers HSPs; ∼22°C induces RBM3; ±0.5°C control is key	Xu et al., 2002; Feng et al., 2022^15^^,^^105^
Schwann cell and neuron co-culture	∼37°C (normothermia)	best Schwann-neuron interaction and myelination at 37°C; transport slows with cooling	slows myelination and proliferation; little benefit; may trigger mild stress	lowers viability and glia-neuron signaling; triggers similar stress as neurons	maintain 37°C; <35°C slows growth; >39°C causes stress; monitor HSPs	Levenstein et al., 2006; Smith et al., 2007^106^^,^^107^
iPSC-derived neurons	∼37°C (normothermia)	differentiation and network form at 37°C; slower synaptogenesis vs. rodent; transport remains normal	improves stress survival; prevents synapse loss in hypoxia; slower growth if prolonged	leads to synapse/cell loss under stress; alters protein expression; triggers HSPs	keep at 37°C; cold induces RBM3, heat induces HSPs; control temp during imaging	Voogd et al., 2024; Gao et al., 2021^108^^,^^109^

### Novel temperature-sensitive proteins in axonal transport

**Figure 5:**
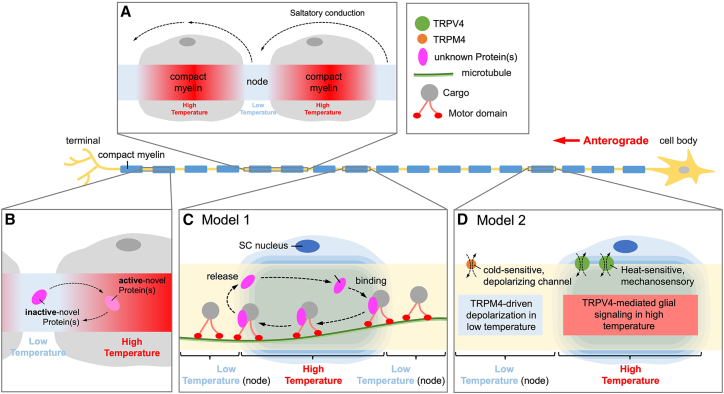
Schematic model of temperature-dependent regulation of protein transport and ion channel function in myelinated axons (A) Illustration of a myelinated axon displaying spatial temperature gradients, with higher temperatures in compact myelin regions and lower temperatures at the nodes of Ranvier (NoRs). This thermal gradient is proposed to facilitate saltatory conduction. (B) Temperature-dependent activation of novel protein(s): inactive proteins in cooler NoR regions transition into an active state within warmer compact myelin zones. (C) Model 1: transport mechanism of novel protein(s): activated proteins are released in high-temperature compact myelin regions, bind to microtubules, and undergo anterograde transport (red arrow) toward the terminal. Proteins may dissociate in cooler NoR regions and recycle back to the cell body or rebind. (D) Model 2: temperature-modulated ion channel distribution: TRPV4 (green) and TRPM4 (orange) channels exhibit region-specific expression and activation, contributing to glial signaling and membrane excitability in response to temperature variations along the axon.

Based on the temperature gradients observed in myelinated axons, we hypothesize that specific proteins regulate axonal transport in response to local temperature variations (Figure 5A). Within myelinated segments, the high temperatures in compact myelin regions may facilitate the activation of novel proteins that promote efficient cargo transport along microtubules. In contrast, the lower temperatures at NoRs may regulate the binding and release dynamics of these proteins, ensuring proper cargo distribution (Figure 5B). We hypothesize that temperature-sensitive proteins act as modulators of axonal transport, either by directly interacting with molecular motors (e.g., kinesins and dyneins) or by influencing the stability of microtubule-associated proteins, with activation occurring in high-temperature compact myelin regions (Figure 5C, model 1). This mechanism would allow motor proteins such as dyneins and kinesins to adapt to temperature variations along the axon, ensuring the continuous and undisturbed transport of essential cellular components.

Although experimental validation is still needed, some known temperature-responsive proteins may act as modulators of axonal transport. For example, heat shock protein 27 (Hsp27/HSPB1) is upregulated during thermal stress and has been shown to stabilize microtubules and regulate axonal integrity under injury conditions.^110^ Similarly, stathmin 1 modulates microtubule dynamics and is regulated by phosphorylation in response to cellular stress, including heat.^111^ Both proteins are expressed in neurons and may influence motor protein processivity or cargo interaction under temperature shifts.^112^^,^^113^ Future studies could explore whether these proteins act as thermosensitive switches in myelinated versus unmyelinated regions by combining localized heat stimulation with proteomics and live imaging.

In summary, technological advancements have greatly facilitated the study of temperature effects on neuronal function. The integration of cutting-edge methods, including gTEMP temperature sensing, super-resolution microscopy, IR-LEGO-based heat stimulation, LC-MS proteomics, and *in situ* electrophysiological recordings, will allow us to test the central hypothesis that temperature differences between compact myelin and NoR modulate motor protein activity and temperature-sensitive proteins. These temperature-dependent mechanisms may regulate cargo transport and saltatory conduction. Together, these approaches offer powerful means to uncover novel thermoregulatory pathways in the nervous system and may inform new strategies for treating disorders involving myelin dysfunction and impaired axonal transport.

### Future directions, challenges, and expected outcomes

While significant progress has been made in understanding how temperature affects axonal transport and SC function in myelinated regions, several key questions remain. In particular, the molecular mechanisms by which thermal gradients regulate protein dynamics, motor protein transport, and ion channel behavior in SCs and axons are still incompletely understood. Our proposed framework (Figures 5C and 5D) highlights two potential models: one involving temperature-dependent activation of novel regulatory proteins (Figure 5C, model 1), and another suggesting region-specific modulation of ion channels such as TRPV4 and TRPM4 in response to temperature shifts (Figure 5D, model 2). In model 1, we hypothesize that newly identified proteins become activated at higher temperatures within compact myelin, facilitating cargo loading or release onto microtubules, while remaining inactive at cooler NoRs. In model 2, TRPV4 and TRPM4 differentially localize and respond to temperature zones, TRPV4 acting as a temperature/mechanosensor in myelinating glia, and TRPM4 modulating excitability near NoRs.

Expected outcomes from our research include he following: (1) mapping intra-axonal and inter-compartmental temperature gradients between the neuron, SCs, compact myelin, and NoRs, and evaluating their effects on conduction fidelity; (2) using high-resolution thermal biosensors (e.g., gTEMP or B-gTEMP), laser microdissection, and LC-MS to identify thermosensitive regulatory proteins specific to myelinated axons; and (3) demonstrating that these proteins exhibit reversible thermal activation and spatial cycling to optimize molecular transport under non-uniform temperature conditions. By coordinating thermal sensing with active transport, these mechanisms may ensure efficient anterograde delivery of cargos while avoiding thermally induced protein dysfunction. Ultimately, uncovering how thermal microdomains regulate glial and axonal physiology will improve our understanding of myelination biology and may reveal novel intervention points for demyelinating and temperature-sensitive neuropathies.

Addressing these questions through future research could provide a more comprehensive understanding of the complex interplay between temperature and the numerous components involved in axonal transport and function in myelinated regions of SCs. Furthermore, elucidating the mechanisms governing temperature-dependent regulation of ion channels, molecular motors, and cytoskeletal components will contribute to our knowledge of the impact of temperature fluctuations on the function of other glial cells, such as astrocytes and oligodendrocytes. Ultimately, a deeper understanding of the role of temperature in neuronal function may have significant implications for the development of novel therapeutic strategies for a wide range of neurological disorders, as well as improving our understanding of the fundamental principles of thermoregulation and its connection to neurological function.

## Conclusions

In conclusion, this review has highlighted the crucial role of temperature in modulating axonal transport and function in myelinated regions of SCs. Our current understanding of the complex interplay between temperature and various cellular processes, such as saltatory conduction, ion channel activity, molecular motor dynamics, Schwann cell function, and the pathophysiology of neurological disorders, is steadily expanding. The emergence of new technologies, such as genetically encoded ratiometric fluorescent temperature indicators, super-resolution microscopy, laser-assisted microdissection, and proteomic analysis, has paved the way for a more detailed investigation of the precise mechanisms by which temperature influences neuronal function. On the other hand, the temperatures introduced in this review are the temperature of the culture medium or the body temperature of the individual, not the temperature distribution at the organelle level in the cell. Therefore, they are merely a guideline, and temperature information with higher spatial resolution is needed for discussions at the molecular level.

The potential implications of our review extend beyond enhancing our understanding of the basic principles of thermoregulation and its connection to neurological function. By unraveling the mechanisms that govern temperature-dependent regulation of ion channels, molecular motors, and cytoskeletal components, we can gain valuable insights into the impact of temperature fluctuations on the function of other glial cells, such as astrocytes and oligodendrocytes. Ultimately, this knowledge may inform the development of novel therapeutic strategies for a wide range of neurological disorders, including MS and CMT disease, where temperature sensitivity has been implicated in disease pathophysiology. As we continue to explore the complex relationship between temperature and neuronal function, interdisciplinary collaboration among researchers from various fields, including neuroscience, biophysics, biochemistry, and computational modeling, will be essential for addressing the remaining challenges and questions. By advancing our understanding of the role of temperature in axonal transport and function, we can contribute to the broader goal of developing novel approaches to improve neuronal regeneration and, ultimately, enhance the quality of life for individuals affected by neurological disorders.

## Acknowledgments

This work was supported by 10.13039/501100001691JSPS KAKENHI grants number 20H02586, 17H06258, 20H05886, 20H05891 and 21K19250 to Y.K., and also supported by the Research Program of “Dynamic Alliance for Open Innovation Bridging Human, Environment and Materials” in “Network Joint Research Center for Materials and Devices” to Y.K.

## Author contributions

C.-W.Z. organized, provided the concept, and created the figure for this manuscript. Both C.-W.Z. and Y.K. contributed to the conception and co-writing of the paper. Y.K. assisted in editing the manuscript and served as the principal investigator in securing funding. Both authors have read and approved the final version of the manuscript.

## Declaration of interests

The authors declare no competing interests.
